# Effect of Moisture on Shape Memory Polyurethane Polymers for Extrusion-Based Additive Manufacturing

**DOI:** 10.3390/ma12020244

**Published:** 2019-01-12

**Authors:** Irina T. Garces, Samira Aslanzadeh, Yaman Boluk, Cagri Ayranci

**Affiliations:** 1 Department of Mechanical Engineering, University of Alberta, Edmonton, AB T6G 2G8, Canada; garces@ualberta.ca (I.T.G.); saslanza@ualberta.ca (S.A.); 2 Department of Civil and Environmental Engineering, University of Alberta, Edmonton, AB T6G2G8, Canada; yaman@ualberta.ca

**Keywords:** shape-memory polyurethanes, extrusion-based additive manufacturing, moisture effects

## Abstract

Extrusion-based additive manufacturing (EBAM) or 3D printing is used to produce customized prototyped parts. The majority of the polymers used with EBAM show moisture sensitivity. However, moisture effects become more pronounced in polymers used for critical applications, such as biomedical stents, sensors, and actuators. The effects of moisture on the manufacturing process and the long-term performance of Shape Memory Polyurethane (SMPU) have not been fully investigated in the literature. This study focuses primarily on block-copolymer SMPUs that have two different hard/soft (h/s) segment ratios. It investigates the effect of moisture on the various properties via studying: (i) the effect of moisture trapping within these polymers and the consequences when manufacturing; (ii) and the effect on end product performance of plasticization by moisture. Results indicate that higher h/s SMPU shows higher microphase separation, which leads to an increase of moisture trapping within the polymer. Understanding moisture trapping is critical for EBAM parts due to an increase in void content and a decrease in printing quality. The results also indicate a stronger plasticizing effect on polymers with lower h/s ratio but with a more forgiving printing behavior compared to the higher h/s ratio.

## 1. Introduction

**Figure 1:**
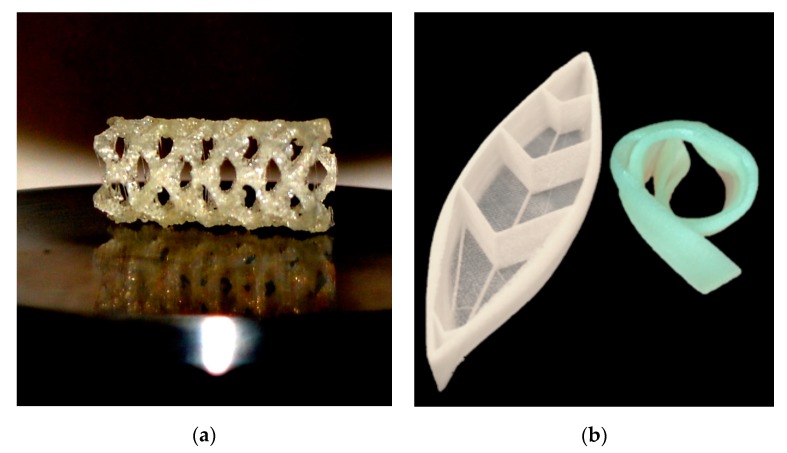
Examples of 3D-printed applications of Shape Memory Polyurethane (SMPU) by EBAM. (**a**) A photograph of an iN–House 3D-printed generic stent and (**b**) 3D-printed kayak prototype based on reference [7].

Over the last decade, extrusion-based additive manufacturing (EBAM), commonly referred to as thermoplastic 3D printing, has gained much attention due to the major advantages it offers for producing customized prototyped parts. To convert EBAM from a prototyping technique to end-use manufacturing that can be utilized for novel and critical applications, it is necessary to learn about some of the difficulties the technology faces. For the majority of the polymers used with EBAM, including polylactic acid (PLA), acrylonitrile butadiene styrene (ABS) and various grades of polyamide (Nylon^®^), moisture is an obstacle during the manufacturing process and later during the part’s life cycle. However, this effect is a more pronounced problem when the printed parts are intended for critical applications, such as in the case of polyurethane-based shape memory polymers (SMPU). It is important to understand the effects of moisture in these materials for EBAM in order to obtain void-less consistent printing quality and predict engineering part performance. Some of the most promising applications of EBAM-based SMPU are biomedical applications such as customized airflow and 3D-printed cardiovascular stents (Figure 1a), casts and splints [1,2,3]. Other critical and promising applications include grippers, actuators for soft robotics [4], origami-based self-assembly structures for smart manufacturing [5,6] and potential printing of smart self-deployable sports equipment such as a shape memory polymer (SMP) 3D-printed kayak [7]. The prototype of a small-scale kayak that may be folded to a temporary shape for storage/transportation and later deployed onsite is seen in Figure 1b.

In general, SMPs are classified into two main categories, those that show a shape memory effect (SME) by means of chemical bonds (commonly in thermosets) and those by means of physical bonds or hydrogen bonding (commonly in thermoplastics) [8]. SMPUs are a type of thermoplastic SMP and many studies have focused in this particular polymer since their commercialization in 1997 [9,10].

SMPU thermoplastics consist of hard-crystallized and soft-amorphous segments [8]. Hard segments commonly have higher transition temperatures—the temperature at which polymeric chains acquire mobility—than do soft segments. This thermodynamic incompatibility causes immiscibility between segments, provoking these two blocks to segregate and produce a microphase separation that leads to an SME [11,12,13]. Within the SME, crystallized segments function as links to the amorphous soft segments [12,14]. Polymeric chains can be set temporarily above the transition temperature and frozen upon cooling. When the polymer is reheated to T > T_transition_, the chains return to their original configuration [15]. 

Different ratios of hard to soft segments (h/s ratio) affect the molecular mobility and determine the properties of a SMPU. This ratio alters the intrinsic properties of the polymer such as its glass transition temperature (Tg), melting temperature (Tm), mechanical properties, shape memory characteristics and effects of moisture on the polymer by plasticization or moisture trapping [12,14]. The individual characteristics of hard and soft segments affect the resulting SMPU. Soft-segments are generally amorphous structures characterized by a low Tg; they are flexible by nature and contribute to the elastic properties of the resulting SMPU [12]. The crystalline structures of hard segments do not change over the range of temperatures used to program the SMPU and act as embedded fillers within the amorphous polymeric structure. In SMPU, urethane segments act as hard segments and polyether or polyester glycols act as soft segments [16,17,18,19,20]. Hydrogen bonding within these hard crystalline structures can potentially serve to set the initial permanent shape [12]. Studies on the chemical, mechanical and shape recovery properties of SMPU have been reported to obtain a better understanding of their properties and functioning [5,21,22,23].

The effects of moisture on SMPUs have been previously studied by investigating the effects of immersion of a thermoplastic SMPU in water. It has been reported that hydrogen bonding, also known as plasticization, alters the Tg and elastic modulus of the SMPU [24,25,26,27,28,29,30]. Plasticization by water lowers the glass transition temperature, mechanical properties and melt viscosity of the SMPU [31,32,33,34,35,36,37,38,39]. An alteration of the glass transition temperature may produce an uncontrolled activation of the SME and undefined mechanical characteristics of the moisture-affected polymer.

Although moisture effects on thermoplastic SMPUs have been studied, investigations have not related moisture effects according to the h/s ratio of different SMPUs and have not linked this to manufacturing technologies such as EBAM. The present study focuses first on the common moisture problem when manufacturing and second on the effect of plasticization by moisture on the chemical, mechanical and shape memory properties of SMPUs and the effects on the 3D-printed part. Understanding the effects of these parameters will significantly increase the use of SMPUs in aforementioned applications. The study outlines an in-depth analysis of moisture effects on two different types of SMPU, one with a low and the other with a higher h/s segment ratio. The study demonstrates the material behavior after a part has been manufactured and the effects of moisture on EBAM manufacturing. 

## 2. Raw Materials

The SMP materials used in this study were purchased from SMP Technologies (Tokyo, Japan). Two different types of ether block copolymer polyurethane thermoplastic SMPs, MM4520 and MM7520, were used. According to the technical sheet provided by the manufacturer, the SMP materials are prepared from diphenylmethane-4,4′-diisocyanate, adipic acid, ethylene glycol, ethylene oxide, polypropylene oxide, 1,4-butanediol and bisphenol A. MM4520 and MM7520 were received in pellet form and have prescribed glass transition temperatures of 45 °C and 75 °C, respectively. 

## 3. Methodology

The methodology is divided into two sections: (i) the effect of moisture trapping in the SMPUs on 3D-printed parts and (ii) a study of the plasticization effect of moisture on two different SMPUs. The latter is relevant to EBAM SMPU parts’ performance under moisture-affected conditions.

For both sections, to avoid any embedded moisture gas bubbles, the SMP pellets were dried for 12 h at 80 °C in a Lindberg/Blue M™ vacuum oven before the melt extrusion processes. Drying time was selected as recommended by the manufacturer and verified by TGA analyses. 

The study of moisture trapping by the SMPU was done on melt-extruded filaments, which were later fed to a commercial EBAM printer to study the effects on printing quality. Studies of moisture absorption characteristics and effects of plasticization by moisture were conducted on melt-extruded SMPU ribbons since this represents the best scenario possible for a 3D-printed part. 

### 3.1. Ribbon Preparation, Compounding and Water Absorption

The samples were prepared by melt-extrusion using a HAAKE^TM^ Haake MiniLab Rheomex CTW5 twin-screw extruder (Waltham, MA, USA). Each screw has a length of 109.5 mm and conical diameters of 5 and 14 mm respectively. The material was poured into a pneumatic feeder and the torque level was adjusted to keep it at a constant 70 N·cm. The extrusion parameters were set to 195 °C and 50 rpm. Ribbons were manufactured using a die with cross sectional dimensions of 4 mm by 0.5 mm. The extruded ribbons were pulled using an LME take up roll system (Dynisco, Franklin, MA, USA) (the diameter of rolls used for the pulling was 1 inch, below the rpm of the roll reported). The pulling speed of the take-up system was kept constant at 9.74 rpm and was measured using an encoder (4096 counts per revolution quadrature encoder, US Digital, Vancouver, WA, USA). 

### 3.2. Filament Preparation for EBAM

To prepare the samples, filaments for the EBAM were melt-extruded using a 3-mm circular cross section die using a Brabender™ single screw extruder attached to an ATR Plasti-Corder drive system (Brabender, Duisburg, Germany). Extrusion parameters for SMPU MM4520 were adapted from Villacres et al. [40], with heating zones of 170 °C, 180 °C, 190 °C and 195 °C, and extrusion rates and pulling speed of 20 rpm. For MM7520, temperatures were set as 175 °C, 185 °C, 195 °C and 205 °C, for the four different heating zones of the extruder. Extrusion rates and pulling speeds were set at 15 rpm.

### 3.3. EBAM Manufactured Samples

Printed (EBAM manufactured) samples were prepared using an Ultimaker™ 2 desktop 3D printer (Ultimaker, Geldermalsen, Netherlands) with a nozzle diameter of 0.4 mm. Printing parameters for MM4520 and MM7520 are portrayed in Table 1. Geometrical parameters such as infill density (100%), infill pattern (±45°) and layer height (0.2 mm) were kept the same for both polymers. Square samples of 30 × 30 mm were printed with an inner central circle of 10 mm and thickness of 3 mm.

## 4. Experimental Methodology

### 4.1. Moisture Manufacturing Effects: Moisture Trapping 

After the extrusion process, filaments were thermally conditioned in an oven and dried at 80 °C for 24 h. Filaments were then immersed in distilled water for different periods. The filaments were immediately fed to the 3D printer and square samples were manufactured. Moisture content after printing was visually and microscopically inspected and compared between both types of SMPU. 

### 4.2. Moisture on Manufactured Parts: Effect of Moisture by Plasticization

After the extrusion process, samples were thermally conditioned and oven dried at Tg + 5 °C for each respective polymer prior to performing water immersion tests. The drying temperatures were chosen to avoid crystallization temperatures of the polymers. To correlate moisture impact on SMPU post-processing to EBAM manufacturing, samples (ribbons) were subjected to 100% moisture content by immersing them in distilled water. Water absorption by the samples was therefore related to the immersion time in distilled water at room temperature. Samples were tested immediately after they were withdrawn from the water. 

#### 4.2.1. Thermogravimetry (TGA) Analysis

TGA samples, weighing 10–15 mg, were cut from the manufactured ribbons and immersed in water for periods of 0, 48, 72, and 112 h at room temperature. TGA analyses were performed to measure the water content of each sample. The analyses were conducted on TA Instruments Q50 Thermogravimetry analyzer (TA Instruments, New Castle, DE, USA). Samples were heated from 25 °C to a maximum temperature of 400 °C at a heating rate 10 °C/min. 

#### 4.2.2. Differential Scanning Calorimetry (DSC) Analysis

DSC tests were conducted using DSC Q1000 (TA Instruments, New Castle, DE, USA). The tests were carried out to examine the potential effect of immersion time (humidity) on Tg. Specimens of 2–5 mg were cut from the manufactured ribbons and were tested using a heating rate of 20 °C/min. The heating rate was chosen to allow comparison with previous studies [27]. The analyses were carried out from −40 °C to 240 °C. Glass transition temperatures were obtained from DSC experimental data. Sample DSC curves of dried polymers are shown in the supplementary information section. The average of three measurements of the glass transition temperatures of the dried polymers were ~44.5 °C and 74.5 °C for MM4520 and MM7520, which is similar to those reported by the manufacturer.

#### 4.2.3. Infrared Spectra Analysis (FT-IR)

Attenuated total reflectance (ATR) was used to collect Fourier transform-infrared spectroscopy (FT-IR) with the aim of recognizing the interaction of water with the polymers. Infrared band analyses were performed using a DIGILAB FTS 7000 Fourier Transform Infrared Spectrometer with UMA 600 microscope (Hopkinton, MA, USA). FT-IR spectra were obtained by averaging 75 scans for each spectrum at a resolution of 2 cm^−1^ in reflection mode. The characterization technique was done on a thin film produced by depositing a layer of 0.4mm of polymer on a heated glass bed using an extrusion-based additive manufacturing desktop printer (Ultimaker ™2+). 

#### 4.2.4. Tensile Testing

Ribbons were cut to 25-mm length samples for testing. A length of 10 mm was left at each side as gripping aids. A final gauge length of 5 mm was left for testing. Tensile tests were conducted using Electroforce^®^ 3200 Series by TA instruments™ (New Castle, DE, USA). Tensile grips were tightened using 17.6 lb-in at each side. The specimens were stretched to 100% in strain using a rate of 0.02 mm/s. Elastic modulus was calculated according to ISO 527-1 for plastics [41]. Due to a high sample elongation and stroke limitation of the tensile device, only elastic moduli were determined for this study.

#### 4.2.5. Shape Recovery Tests

For MM4520, we tested seven samples at 0, 12 and 24 h each. For MM7520, we tested seven samples at 0 and 24 h each. Samples were cut to 45-mm lengths. Gripping lengths of 10 mm were used, leaving a final gauge length of 25 mm for testing. The gauge length was chosen due to the limitation of the machine’s stroke. The shape programming was done using Electroforce^®^ 3200 Series by TA instruments ™ equipped with a heated forced-air convection chamber. 

**Figure 2:**
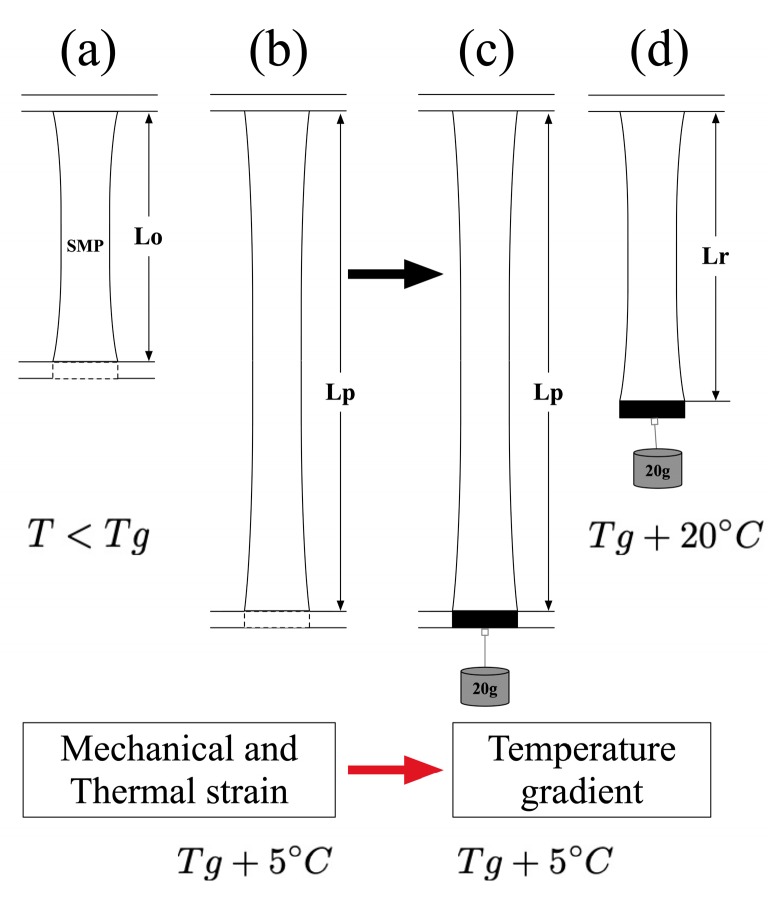
Schematic showing the programming and shape recovery procedure of a Shape Memory Polymer ribbon, where (**a**) shows the original sample (**b**) and (**c**) the sample while and after shape programming, respectively and (**d**) the recovered sample.

Figure 2 shows the schematic representation of the programming cycle done on SMP specimens. The surrounding temperature was raised from room temperature to 50°C (Tg + 5 °C) for MM4520 samples and to 80 °C (Tg + 5 °C) for MM7520 at a heating rate of 0.50°C/sec during 3 minutes. To obtain an optimal recovery and allow for the polymer to store energy, a programming temperature of 50 °C was chosen for MM4520, similar temperatures have been considered in previous studies for materials having a glass transition temperature close to that mentioned previously [27,29,42]. Therefore, a temperature of 80 °C was chosen for MM7520.

Figure 2a shows a sample fixed at both ends. From Figure 2a to Figure 2b, the sample was stretched to 100% strain, from the original length (Lo) to the prescribed length (Lp), at a rate of 0.2 mm/s. Immediately after, the sample was cooled down to Tg using forced air to prevent any further recovery. The samples were then placed in a cooler until activation.

The activation, or recovery, process was conducted in a Lindberg/Blue M™ vacuum oven (Waltham, MA USA) set to 70 °C (Tg + 25) for MM4520 samples and to 100 °C (Tg + 25) for MM7520 samples. During activation, a small constant load, corresponding to ~0.2 N, was applied at the free end of the sample to prevent curling during recovery. Samples recovered their shape from Figure 2c to Figure 2d. For analysis, pictures were taken at 4 Hz for 5 min during activation and change in length was measured according to [43]. 

## 5. Results and Discussion 

### 5.1. Moisture Manufacturing Effects: Moisture Trapping 

**Figure 3:**
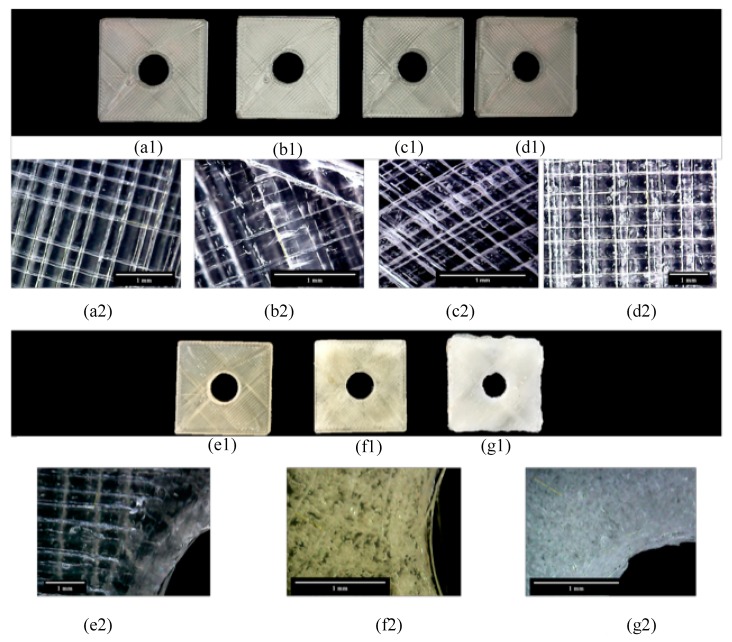
Photographs showing the effects of moisture in filaments on EBAM manufactured parts. Figure (**a1**), (**b1**), (**c1**) and (**d1**) are images of MM4520 printed parts manufactured with filaments immersed in water for 0, 10 min, 5 h and 24 h. Figures (**a1**), (**b2**), (**c2**) and (**d2**) show zoomed-in views of the printed surfaces. Similarly (**e1**), (**f1**) and (**g1**) are images of MM7520 printed parts manufactured with filaments immersed in water for 0, 10 min and 24 h, the close-up versions are shown in (**e2**), (**f2**) and (**g2**).

Moisture trapping within SMPUs is significant for any melt-extrusion manufacturing process. Figure 3 shows photographs of the outcome of 3D-printed SMPU parts when the material is affected by moisture prior to the EBAM process. Figure 3(a1,b1,c1,d1) are images of MM4520 printed parts manufactured with filaments immersed in water for controlled moisture exposure (0 min, 10 min, 5 h, and 24 h). From afar, printed part quality is not affected by moisture absorption of the filaments. Figure 3(a2,b2,c2,d2) show zoomed-in views of the printed surfaces. These images show an increase of imperfections of the surface (circle like patterns), which indicates moisture trapping before reaching water saturation. These imperfections are clear voids within the 3D-printed structure. There is a gradual increase in the number of voids in the specimen from the dried specimen to the moisture-saturated specimen. 

Figure 3(e1,f1,g1) are images of MM7520 printed parts manufactured with filaments immersed in water for 0, 10 min and 24 h. The printed part quality decreases significantly with respect to moisture affected filaments. The effects can be seen by a color and surface change from uniform transparent to white rough surfaces. This whiter surface has a high percentage of voids, as can be closely seen in Figure 3(e2,f2,g2). The images show that moisture trapping increases dramatically after only 10 min of water absorption. 

### 5.2. Moisture on Manufactured Parts: Effect of Moisture by Plasticization 

#### 5.2.1. Moisture Absorption Content

**Figure 4:**
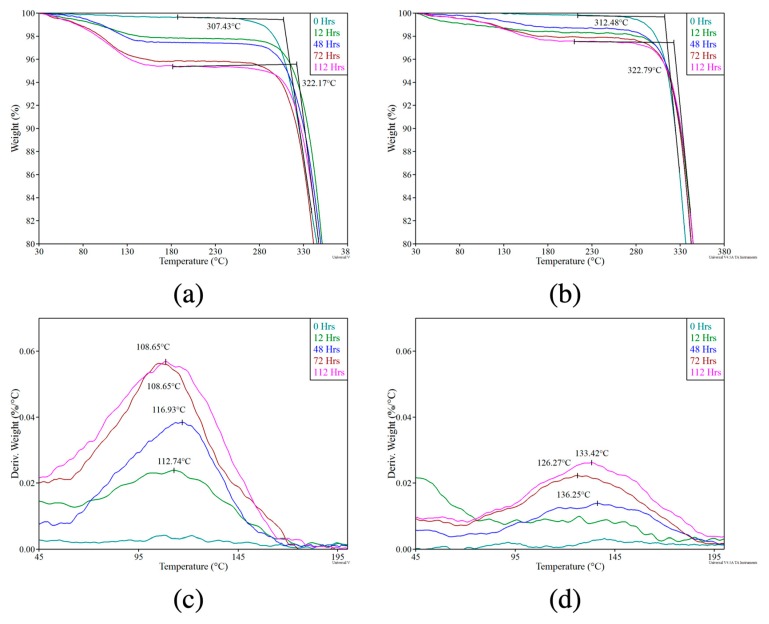
TGA and DTGA curves of (**a**) and (**c**) MM4520; and (**b**) and (**d**) MM7520 after different immersion times in water.

The TGA plots of moisture-saturated materials, MM4520 and MM7520, are presented in Figure 4a,b, respectively. The tests were done with samples that were first immersed in water for 0, 48, 72, and 112 h. Both the polymer matrices absorbed water and reached a saturation point after 72 h. Figure 4a,b also show the degradation temperature (Td). Both of the polymer samples started to decompose at ~300 °C. Therefore, thermal degradation of the polymers was independent of the absorbed water content. The most water evaporation (removal) occurs before 48 h, as seen by the greatest weight loss percentage in the TGA. After 72 h, weight loss gradually decreased and eventually stabilized. 

Figure 4c,d show the derivative thermogravimetric analysis (DTGA) for MM4520 and MM7520, respectively. These were obtained by taking the derivatives of the data presented in Figure 4a,b with respect to temperature (i.e., zoomed in between 45 and 200 °C). The peak represents the point of maximum moisture removal (in Figure 4c for MM4520). The peak values for the various immersion times of samples are shown in Figure 4c for MM4520. As the immersion time of the polymer in water increased, the maximum point of the rate of desorption shifted to a lower temperature by a few degrees. On the other hand, no visible desorption trend found in MM7520 with various water immersion times (Figure 4d). This data suggests that a greater amount of water was absorbed and free water moved more readily from MM4520 than MM7520. This is especially interesting for drying purposes since a drying procedure will have to be performed on 3D printing filaments. Yang et al. also suggested that free water has a negligible effect on Tg and can be fully removed below 100 °C, whereas bound water directly affects Tg. Bound water can be removed by heating above 120 °C [27]. Because of the nature of the EBAM process, thermal history will be wiped after melting the material, therefore drying conditions for EBAM filaments can include bounded water removal temperature ranges.

**Figure 5:**
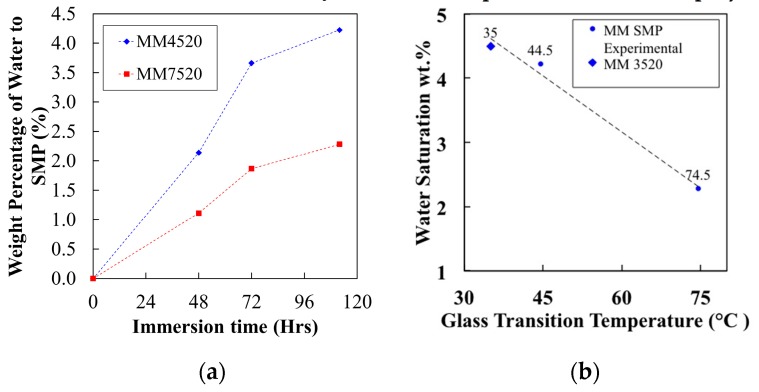
(**a**) Ratio of water content to MM4520 and MM7520 after different immersion times in water (**b**) water Saturation with respect to Shape Memory Polyurethane Tg series.

Figure 5a shows the water content (weight %) of MM4520 and MM7520 samples as a function of their immersion time in water. Plots were generated by calculating the water content values from Figure 4a,b data at around 240 °C, assuming that all weight loss was due to the evaporation of water and that the polymer became completely free of moisture. The plots indicate that MM4520 contained 4.22% moisture intake compared to 2.28% for MM7520 at saturation. The differences between SMP samples are due to the different amounts of soft segment content of each polymer.

Figure 5b shows the plot of water saturation values vs. glass transition temperatures of the SMPU samples. In addition to our measurements of MM4520 and MM7520, the data for MM3520 was compiled from the literature [27]. Glass transition temperature has been reported to increase linearly with respect to hard segment content [17]. A linear relationship of total moisture saturation can be related to the ratio soft/hard segments, or the glass transition temperatures of SMPU samples; thus, the soft segments of the SMPU can be directly associated to plasticization in the polymer matrix. 

#### 5.2.2. Moisture and Its Effect of Hydrogen Bonding (Plasticization)

The literature covers extensively the matter of hydrogen bonding in SMPUs due to moisture absorption [24,26,27,29,44,45,46].This phenomenon can be characterized by observing a wave number shift of C=O and N–H stretching to lower values from the original non-interacted peaks [44,45,46]. The Infrared Spectroscopy for (a) MM4520 and (b) MM7520 can be found in the Supplementary Information. Comparative values of the peaks of dried and water-immersed samples are summarized in Table 2. 

The intrinsic properties of shape memory polymers, such as shape recovery and mechanical properties, are closely related to their degree of phase separation $\left(D_{ps}\right)$. The intermolecular hydrogen bonding between the carbonyl group (peak at ~1700 cm^−1^) and the NH group, from the urethane hard segment, can be used to determine the degree of phase separation. Merline et al. [17] suggested that the degree of phase separation can be calculated through (Equation (1)). The comparison is thus considered between the bonded absorption bands $\left(A_{b}\right)$ and free absorption bands $\left(A_{f}\right)$ of the carboxyl groups.

(1) Dps=(Ab/Af)1+(Ab/Af)

**Figure 6:**
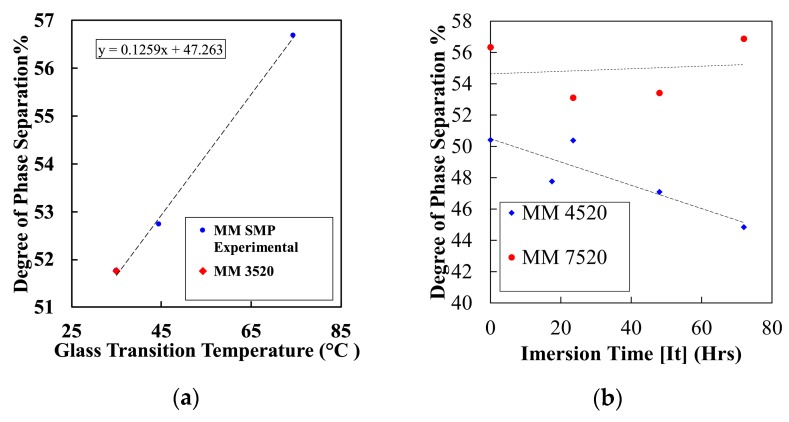
(**a**) Degree of phase separation with respect to different SMPU series (MM3520, MM4520 and MM7520) and (**b**) change in the degree of phase separation with respect to moisture absorption for MM4520 and MM7520.

From the obtained calculation, Figure 6a shows a plot of the calculated degree of phase separation for three different commercially available polymers, MM3520, MM4520 and MM7520. Data for MM3520 was obtained from [27]. The figure shows a linear increasing trend between the calculated degrees of phase separation as glass transition temperature of SMPU increases. Because the glass transition temperature is closely related to the amount of hard segment content within the polymer, it can be directly inferred that an increase of hydrogen bonding between hard segments occurs [27]. Wen et al. [46] also showed that there exists a proportional relation between hard to hard hydrogen bonding and phase separation between soft and hard segments for thermoplastic polyether-based polyurethane (N–H⋯O=C bond). Thus, the degree of phase separation plays a significant role in determining the shape memory characteristics of the material.

Studies have revealed that water has a direct effect on hydrogen bonding within the SMPU’s structure. It has been suggested that water molecules act as bridges between hydrogen-bonded NH and C=O groups [29]. The degree of phase separation has also been determined with respect to absorbed moisture by the polymers (i.e., immersion time in water). Figure 6b shows the variation of degree of phase separation with respect to immersion time in water for MM4520 and MM7420. There is a clear decrease in phase separation for MM4520 but no visible effect for MM7520. This confirms that hydrogen bonding decreases phase separation by bridging soft and hard segments.

**Figure 7:**
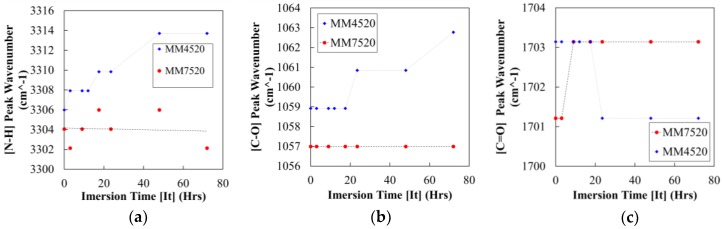
Values of bond stretching for (**a**) N–H bond, (**b**) C=O and (**c**) C–O bonds.

From the FT-IR results, bond stretching (a) N–H (b) C–O and (c) C=O for MM4520 and MM7520 were studied after different immersion times in water. An increase in wavenumber was observed from (N–H⋯H) and (C–O⋯H) groups for MM4520 in contrast to MM7520 (Figure 7a,b). These results show a weakening of hydrogen bonding for MM4520 as water absorption occurs [29], [47]. The loosely bound water weakens the hydrogen bond and decreases Tg. A weakening of hydrogen bonding is also related to a phase separation between soft and hard segment, as suggested by Wen et al. [46].

Hydrogen bonded C=O shows a slight increase for MM7520 and a slight decrease in MM4520 (Figure 7c), this result is in agreement with Yang’s [29] study of MM3520. Yang showed that within the chemical structure of SMPU MM3520 hydrogen bonding is present at two points of the C=O group. The two hydrogen bonds provoke a counter reaction, forcing the infrared band peak to decrease [29]. Because of the close glass transition temperatures between MM3520 and MM4520, the same phenomena can be inferred for MM4520. Contrary to MM3520 and MM4520, the infrared band on the C=O group for MM7520 increases, suggesting that there is only one place at which bonded water occurs. Studies have shown that bounded water affects the shape fixity and shape recovery of SMPUs and directly affects their glass transition temperature. Free water, on the other hand, has a negligible effect [24,26,27]. We conclude that, in contrast to MM7520, MM4520 shows more bound water and presents a visible decrease in phase separation; moisture will have a more severe effect on MM4520 than on MM7520.

#### 5.2.3. Moisture Absorption and Glass Transition Temperature 

**Figure 8:**
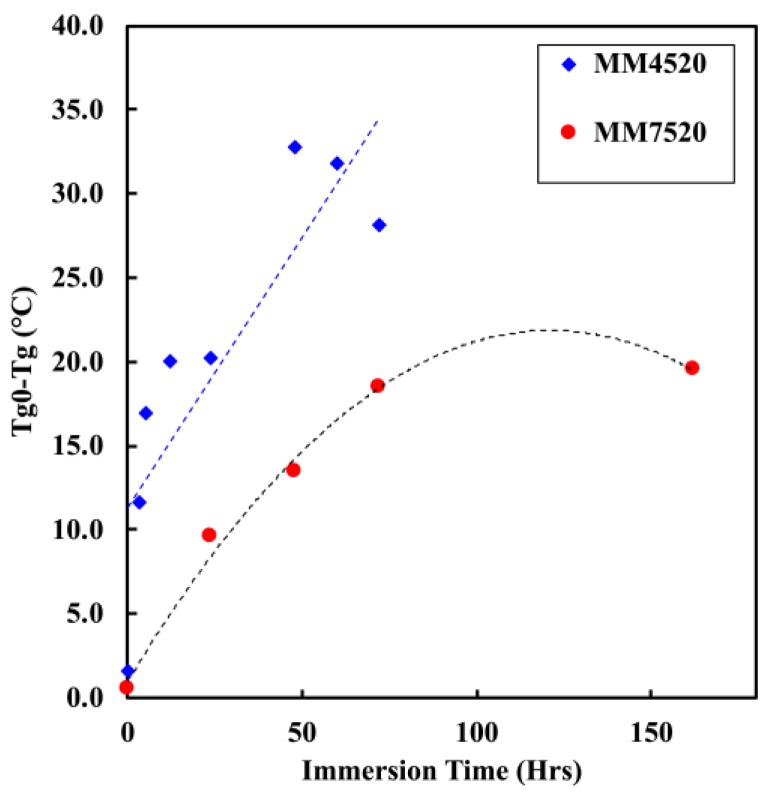
Graph showing a change in Tg with respect to immersion time in water for MM4520 and MM7520.

Figure 8 shows the change and decrease in glass transition temperature (Tg) for MM4520 and MM7520 as the immersion time in water increases. Previous studies have shown that moisture has a plasticization effect on thermoplastic polyurethanes causing a decrease on the glass transition temperature [24,26,27,35]. Yang et al [27] showed that bound water reduces the glass transition temperature in a linear manner. On the other hand, free water has been shown to have a negligible effect on the glass transition temperature and mechanical behavior, as expected from the degree of phase separation in Figure 6b. Figure 8 shows a rapid change in Tg for MM4520, while the decrease of Tg for MM7520 is much slower, with a faster saturation. The Tg decrease of MM4520 is similar to the decrease of MM3520 found by Yang et al. [27]. We can see that plasticization by water occurs in both polymers but is more prominent for MM4520 than for MM7520. This is possibly due to a higher bound-to-free water ratio in MM4520 than in MM7520, when in contact with water. 

#### 5.2.4. Moisture Absorption and Mechanical Properties 

**Figure 9:**
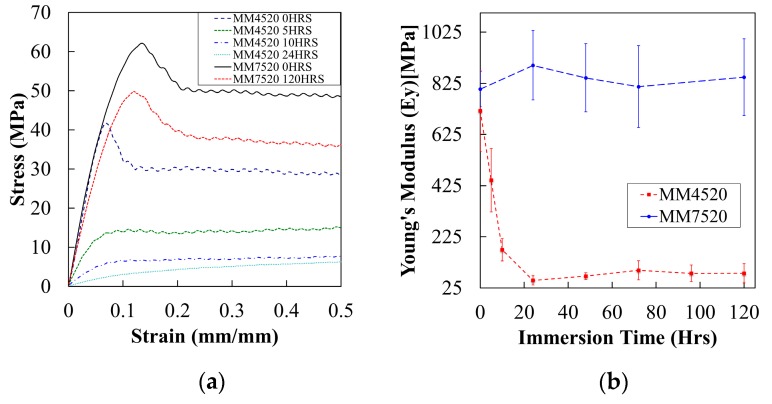
Graphs showing the mechanical behavior of both materials. (**a**) Describes the evolution in mechanical behavior while (**b**) shows the drop in elastic modulus with respect to immersion time in water.

Of all the materials tested, the effect pf moisture on mechanical properties is the most significant for MM4520. Figure 9a shows the evolution of mechanical behavior for MM4520 and MM7520. MM4520 changes from a tough material with a defined yield point to a material without a clear yield point when immersed in water (see plots corresponding to legends MM4520 0/5/10/24HRS). Nevertheless, almost no significant effect on MM7520 was observed (see plots corresponding to legends MM7520 0/120HRS). This is caused by the lubrication of chains and a decrease of phase separation due to plasticization, which improves the elasticity of the material.

Figure 9b shows the elastic modulus (*E_y_*), and one standard deviation at each side, obtained from tensile tests conducted for MM4520 and MM7520 with respect to immersion times in water. The elastic modulus of dried MM4520 (717 MPa) and MM7520 (803 MPa) did not differ significantly. Additionally, a rapid decrease of the elastic modulus for MM4520 was observed as immersion time in water increased. There was no significant effect for MM7520. The obtained difference between both polymers is given by a higher plasticization of the polymer with a lower hard-to-soft segment ratio (MM4520) compared to MM7520, just as it was seen by Yang et al. on a polymer with lower hard-to-soft segment ratio [27,29].

#### 5.2.5. Model Description of MM4520 Elastic Modulus and Tg Relationship

Because of plasticization effect using a low Tg is necessary in many engineering and biomedical applications, such as body temperature-activated devices, including stents. Therefore, an understanding of the material’s behavior is necessary to predict product performance. In the present study a model is proposed to predict the mechanical properties, with respect to moisture content, of a material with a lower Tg. Reimschuessel et al.’s logarithmic model [38] is used to obtain, via a regression analysis, an approximate solution of MM4520’s uniaxial elastic modulus with respect to immersion time in water. Reimschuessel et al. described a decreasing logarithmic model that shows the relationship between the elastic modulus and water content percentage for the plasticization effect in polymers [38]. Equations were adapted from Reimschuessel et al. [38] model.

The experimental data shows that the modulus (*Ey*) of MM4520 decays with respect to immersion time in water, reaching a saturated Elastic Modulus $\left(E_{S}\right)$. The initial elastic modulus for water content at 0 h is described by $\left(E_{o}\right)$, where immersion time in water is W(0)=0. Therefore, the boundary condition is as follows $\left(E_{y}\left(0\right)=E_{o}\right)$.

(2) $$\frac{dE_{y}}{dW}=-k_{\left(E_{o},E_{s},W_{s}\right)}\cdot \left(E_{y}-E_{s}\right)$$

(3) $$E_{y}=(E_{o}-E_{s})e^{-k\cdot W}+E_{s}$$

(4) $$k_{\left(E_{o},E_{s},W_{s}\right)}=\frac{\ln\left[\frac{E_{o}-E_{s}}{E_{s+1}-E_{s}}\right]}{W_{s-1}}=\frac{\ln\left[E_{o}-E_{s}\right]}{W_{s}-\delta_{w}}=\ln\left[E_{o}-E_{s}\right]\eta /W_{s}$$

(5) $$E_{y}=(E_{o}-E_{s})e^{-\left(\ln\left[E_{o}-E_{s}\right]\eta /W_{s}\right)\cdot W}+E_{s}$$

In this model, the elastic modulus decreases to a finite value $\left(E_{s}\right)$ after an amount of water, indicated as $W_{s},$ has saturated the polymer. This is identified as $Ey_{\left(W_{s}\right)}=E_{s}$ and therefore $\frac{dE_{y}}{dW}=0,\;W_{s}\to \infty$. The speed at which the modulus decreases, described mathematically by *k*, is a function of $E_{o},E_{s}$ and $W_{s}$ (Equation (2)). The solution to the ordinary differential equation and its constant for the initial boundary condition results in Equation (3)). To write this in terms of a saturation time or water saturation content, W, we define a value of 1 as an artifice, where $E_{s+1}-E_{s}=1$. Consequently, $W_{s-1}-W_{s}=\delta_{w}$, and, using $\eta =\frac{W_{s}}{W_{s}-\delta_{w}}$, we obtain an expression for constant k.

**Figure 10:**
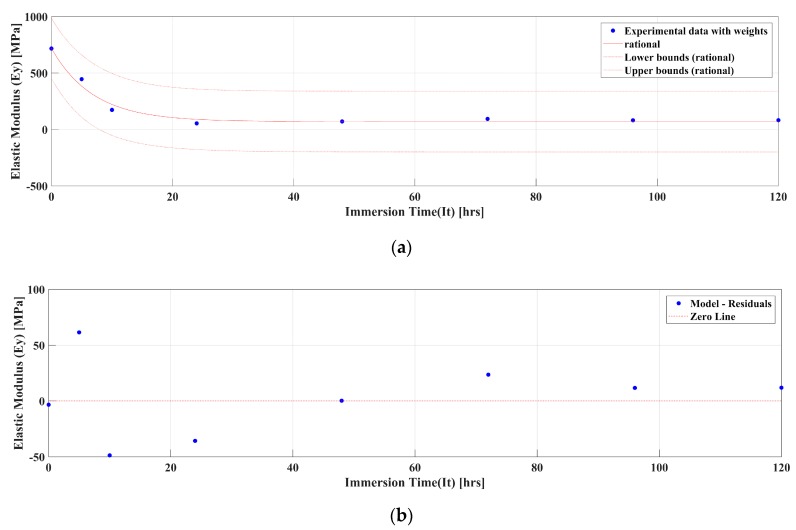
Graph of elastic modulus (*Ey*) for MM4520 for (**a**) fitted model and (**b**) residuals.

Using a nonlinear least squares fit in MATLAB^TM^, the experimental data was weighted with respect to the number of experiments conducted and fitted to find the coefficients of the following model $f\left(x\right)=a_{1}e^{-\frac{x}{b_{1}}}+c_{1}$, which is in the same form as equation (Equation (5)). Coefficients were obtained with 95% confidence intervals and their values are portrayed in Table 3. Figure 10a shows the obtained Young’s modulus from the experimental data, model curve fit and statistical lower and upper bounds. Regression residuals for MM4520 are presented in Figure 10b. The curve fit results in an adjusted coefficient of determination of $R^{2}=0.9895$, showing that Reimschuessel et al.’s model correlates with our experimental data. For the predicted model, water saturation is considered at 99% of water saturation value, with a resolution of 0.01 h. of immersion time in water. The final generic model shown in Table 3 was calculated from the obtained curve fit model coefficients. Without any more experiments, the elastic modulus can be predicted with respect to immersion time in water for SMP MM4520. Similarly, the same procedure can be applied to determine the glass transition temperature of both materials. However, a statistical model will be more significant with a higher sample size that the one obtained in this study.

#### 5.2.6. Moisture Absorption and Shape Recovery Properties

**Figure 11:**
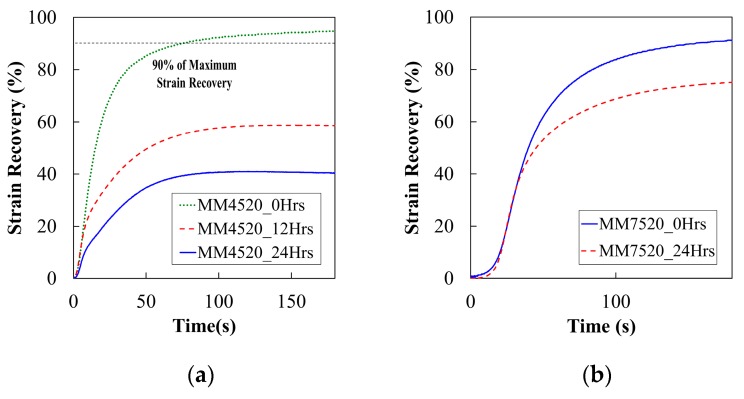
Representation curves for strain recovery of (**a**) MM4520 at activation temperature T = 50 °C and (**b**) MM7520 after different immersion times in water at activation temperature T = 80 °C.

To observe the possible effects of moisture in the strain recovery process, we performed a one free end recovery with an application of 0.2 N in force. The strain recovery percentage, portrayed in Equation (6), was obtained from the ratio of the difference between the prescribed strain ($\varepsilon_{p}=100\%$) and the recovered strain ($\varepsilon_{r}$) to the original prescribed strain [18]. Figure 11a,b, respectively, show the recovery for samples of MM4520 at 0, 12 and 24 h and 0 and 24 h for MM7520. The longer the samples were in water, the less able they were to recover, as can be seen in the figures. Recovery percentage was measured at 90% of the maximum recovery’s plateau. See Figure 11a.

(6) $$R_{r}=\left(\varepsilon_{p}-\varepsilon_{r}\right)/\varepsilon_{p}$$

**Figure 12:**
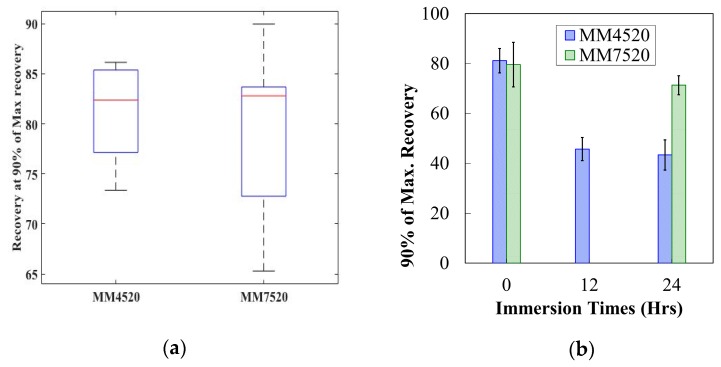
(**a**) Box plot showing recovery of MM4520 and MM7520 dried materials and (**b**) maximum recovery of MM4520 and MM7520 after water immersion.

The tests showed that both materials recovered the same amount after being activated (Figure 12a). As moisture percentage increased, 90% of the maximum recovery decreased significantly for both materials, as portrayed in Figure 12b. The ANOVA returned a *P*-value = 1.992 × 10^−10^ < 0.05 for MM4520 and a *P*-value = 0.027 < 0.05 for MM7520. The amount of recovery found is less than what has been reported [48]. This may be caused by the applied strain and the additional force (0.2 N) placed at the free end of the samples. However, the procedure was kept constant allowing a comparison of the relative values for water-immersed samples and dried. The effect on glass transition temperature may have an effect on the programming temperature and the ability of the material to recover to a maximum strain. The programming temperature does not change. Therefore when these polymers reach a temperature where plastic deformation occurs, the polymer loses the ability to store elastic stress [49].

**Figure 13:**
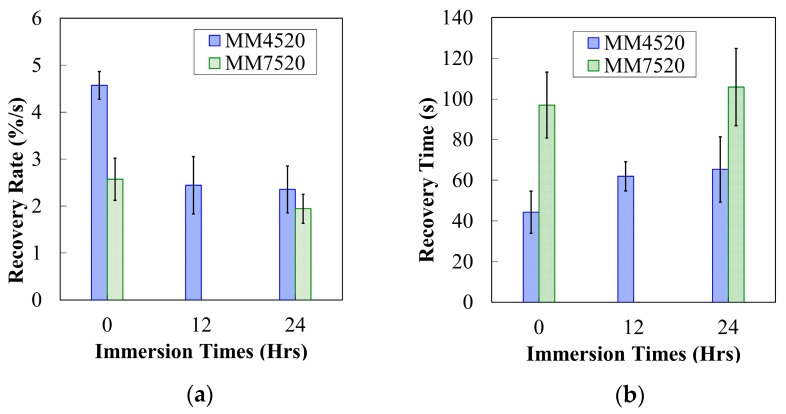
(**a**) Recovery rate for MM4520 and MM7520 with respect to immersion times in water (**b**) Recovery time for MM4520 and MM7520 with respect to immersion times in water.

The required time to reach 90% of the maximum recovery’s plateau was also recorded. Recovery rate for both materials was obtained from the slope of the strain recovery percentage with respect to time. Figure 13a shows the recovery rate with respect to time for MM4520 and MM7520. P-values obtained for MM4520 and MM7520 were 8.47 × 10^−8^ and 0.0070, respectively, showing a significant difference in recovery rate for both materials. Because both materials lose their elastic capacity when being programmed, their ability to recover at a fast rate is also affected, just like a spring would be when being deformed. Consequently, a trend of increasing required time with an increase in moisture content is observed in Figure 13b. *P*-values of 0.0123 and 0.9231 for MM4520 and MM7520 were obtained, respectively, showing a significant effect of moisture on recovery time for MM4520 while no significant effect was found for MM7520. 

Effects of moisture on recovery rate and time may be caused by the evaporation of bounded and free water molecules within the samples. It is noted that free water is easier to evaporate than bounded water [26,50]. Because of its higher hard-segment to soft-segment ratio, the recovery of MM7520 is not as affected by moisture as MM4520. Therefore, it is able to evaporate moisture content quicker because it is harder to produce hydrogen bonds within its structure. 

## 6. Conclusions

We investigated the influence of moisture on the preprocessing stage of manufacturing and in the post manufacturing stage of two SMPUs containing hard to soft segment ratios. For the preprocessing stage, filaments of SMPU were manufactured and immersed for different periods in distilled water and immediately fed into a commercial 3D printer. The results showed that the polymer with higher Tg (higher h/s ratio) trapped more moisture, affecting dramatically the printing quality only 10 min after moisture exposure. This phenomenon is inferred to have occurred due to water molecules trapped within the phase separation that produce voids when evaporated. 

The degree of phase separation increases as the transition temperature (Tg or h/s ratio) of the SMPUs increases. As the degree of phase separation increases, there exists a wider space for water molecules to be stored and create voids when the polymer is being heated and extruded during EBAM. This is clearly seen in the printed qualities of the two SMPUs. Although moisture content is related to water immersion, effects of moisture were also seen in filaments exposed to ambient conditions for less than 48 h (with uncontrolled moisture exposure). 

SMPUs with higher h/s segments (higher Tg), such as MM 7520, are more susceptible to moisture trapping. The final quality of a melt-extruded part is severely affected by moisture in polymers with a higher Tg. Although a higher h/s segment ratio is more affected by moisture trapping, because of its resistance to hydrogen bonding and therefore plasticizing, these SMPUs are more beneficial for manufacturing. To obtain void-less printed parts and therefore a better printing quality when using an SMPU with a higher Tg for 3D printing, rigorous precautions must be taken to maintain the dry conditions of the printing filaments.

Although the polymer with higher Tg was more affected in the preprocessing stage, in the post-manufacturing stage the material was less affected by plasticization effects. The polymer with the lower Tg was more affected by the formation of hydrogen bonding into (N–H) and (C–O) groups when immersed in water. This is caused by a lower hard-to-soft segment ratio in the polymer with a lower Tg. This study also showed that, due to plasticization, moisture absorption has a more severe effect on the mechanical properties of the material with a lower Tg. The elastic modulus slowly reached a saturated modulus for the polymer with lower Tg, while there was no visible effect on the polymer with the higher Tg. From these observations, a generic model for predicting the elastic modulus with respect to immersion time was obtained for an SMP with a 45 °C Tg. A similar decreasing exponential effect was observed for the mechanical properties on the polymer with nominal 45 °C Tg. A linear decreasing trend was observed on the other polymer. 

Moisture also had a significant effect on the shape recovery properties of the material. The ability to reach 90% of the maximum recovery and the recovery rate for both materials decreased when the material was immersed in water. This may be caused by the plastic deformation that the polymeric chains suffer from the decrease of Tg and the applied strain. Moisture did not have a significant effect on the time required to reach 90% of the maximum recovery on the polymer with a higher Tg, but had a significant effect on the polymer with a 45 °C Tg. This may be caused by the evaporation time for free water in contrast to bound water. Since the polymer with the higher Tg has a higher hard-segment ratio, it can be inferred that it will have less bounded water than the polymer with lower Tg. 

An SMPU with a higher Tg, which has a higher h/s segment ratio, resists hydrogen bonding and therefore plasticization. This makes it more desirable for ambient moisture exposure applications. However, due to a higher degree of phase separation, moisture trapping has a significant effect when subjected to melt extruded manufacturing techniques. This effect becomes critical in EBAM-3D printing manufacturing. The present study contributes information for engineers and designers regarding the material selection for shape memory polyurethane designs for emerging manufacturing technologies such as 3D printing. 

## Figures and Tables

**Table 1:** Printing parameters used when 3D printing with each respective material (MM4520 and MM7520).

Material	Nozzle Temperature	Printing Speed (mm/s)	Build Plate Temperature	Fan Speed
MM4520	210	10	50	10%
MM7520	230	10	80	10%

**Table 2:** Bonding frequencies for dry materials.

Material	FTIR -Wavenumbers [cm^−1^]				
	Bonded C=O	Free C=O	Bonded N–H	Free N–H	Bonded C–O
MM4520	1703	1724	3306	3400	1059
MM7520	1701	1730	3304	3383	1057

**Table 3:** Estimated coefficients for MM4520 elastic modulus with respect to immersion time in water.

Estimation curve:	$f\left(x\right)=a_{1}*e^{-x/b_{1}}+c_{1}$			
Estimated Coefficients:	$a_{1}\;$ (95% CI)	$b_{1}$ (95% CI)	$c_{1}$ (95% CI)	
	650	6.888	70.4	
Final generic model:	$E_{y}=(E_{o}-E_{s})e^{-\left(ln\left[E_{o}-E_{s}\right]\eta /W_{s}\;\right)\cdot W}+E_{s}$			
Estimated Constants:	***Eo*** (**MPa**) (95% CI)	***Es*** (**MPa**) (95% CI)	***Ws*** (**h**) (95% CI)	***η***1**MPa** (95% CI)
	720.4	70.4	31.17	−0.698

## Supplementary Materials

The following are available online at http://www.mdpi.com/1996-1944/12/2/244/s1, Figure S1: FTIR spectrums for (a) MM4520 and (b) MM7520 with zoomed in at different bonds title, Figure S2: Examples of DSC measurement for MM4520 and MM7520.
