# Supplementary material for: Effect of Moisture on Shape Memory Polyurethane Polymers for Extrusion-Based Additive Manufacturing
Source: Materials (Basel). 2019 Jan 12;12(2):244. doi: 10.3390/ma12020244 (PMC6356422; doi:10.3390/ma12020244)
Supplement: Supplementary file 1 [file materials-12-00244-s001.pdf]

Supplementary Information

1. FTIR and DSC

In Figure 1.a and b, hydrogen bonded groups appear as strong peaks while free groups appear as slight shoulders. Free groups for MM4520 and MM7520 appear in a wavenumber of ~3400 cm^{-1} and 3383 cm^{-1} for N-H, respectively; and for free C=O appear approximately about ~1724 cm^{-1} and 1730 cm^{-1} respectively. Similar studies have been performed on SMP MM3520 by Yang et al. [1], and values found are in agreement to those discussed by the authors and other studies performed on thermoplastic polyurethanes [2]-[5].

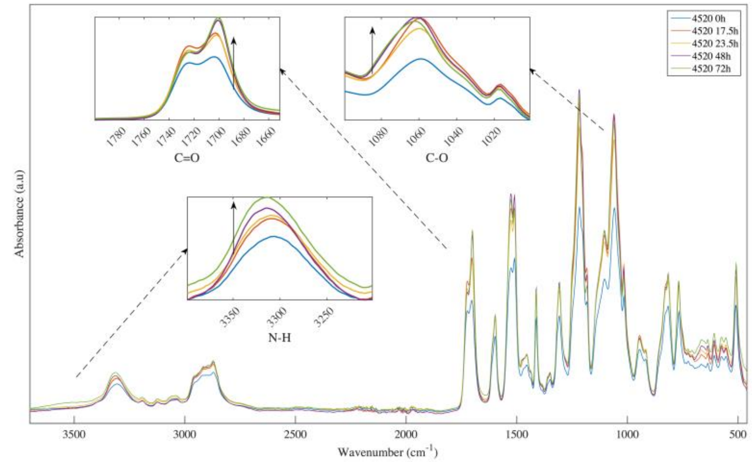

(a)

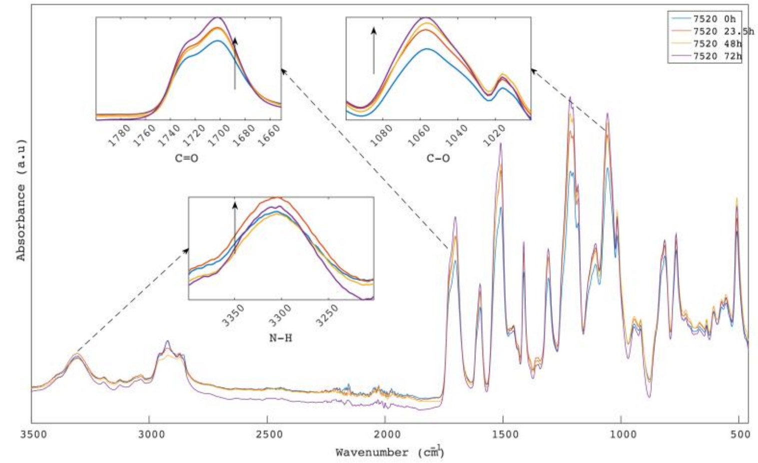

(b)

Figure 1. FTIR spectrums for (a) MM4520 and (b) MM7520 with zoomed in at different bonds

Sample measurements of DSC are reported on Figure 2 for MM4520 and MM7520. Samples were also verified via Modulated DSC, samples are similar to those found by the manufacturer.

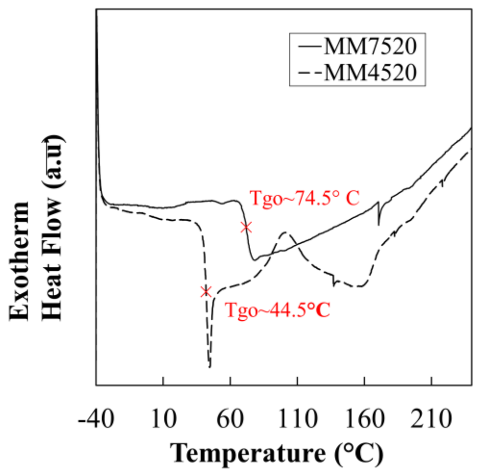

Figure 2. Examples of DSC measurement for MM4520 and MM7520

References

[1] B. Yang, "Influence of moisture in polyurethane shape memory polymers and their electrically conductive composites.," 2007.

[2] T.-C. Wen, M.-S. Wu, and C.-H. Yang, "Spectroscopic Investigations of Poly(oxypropylene)glycol-Based Waterborne Polyurethane Doped with Lithium Perchlorate," *Macromolecules*, vol. 32, no. 8, pp. 2712–2720, Apr. 1999.

[3] C. Zhang, "Computational study of hydrogen-bonding interactions in shape memory polymers," 2014.

[4] C. M. Brunette, S. L. Hsu, and W. J. MackKnight, "Hydrogen-Bonding Properties of Hard-Segment Model Compounds in Polyurethane Block Copolymers," *Macromolecules*, vol. 15, no. 1, pp. 71–77, 1982.

[5] E. Yilgor, I. Yilgor, and E. Yurtsever, "Hydrogen bonding and polyurethane morphology. I. Quantum mechanical calculations of hydrogen bond energies and vibrational spectroscopy of model compounds," *ADDMA*, vol. 43, no. 24, pp. 6551–6559, Nov. 2002.
